# Barriers to surgical care in Nepal

**DOI:** 10.1186/s12913-017-2024-7

**Published:** 2017-01-23

**Authors:** Joris Adriaan Frank van Loenhout, Tefera Darge Delbiso, Shailvi Gupta, Kapendra Amatya, Adam L. Kushner, Julita Gil Cuesta, Debarati Guha-Sapir

**Affiliations:** 1 Centre for Research on the Epidemiology of Disasters, Université Catholique de Louvain, School of Public Health, Clos Chapelle-aux-Champs 30, 1200 Woluwé-Saint-Lambert, Brussels, Belgium; 2 Department of Surgery, University of California San Francisco East Bay, Oakland, CA USA; 3 Department of Surgical Oncology, Nepal Cancer Hospital, Kathmandu, Nepal; 4 Department of International Health, Johns Hopkins Bloomberg School of Public Health, Baltimore, MD USA; 5 Department of Surgery, Columbia University, New York, NY USA; 6 Surgeons OverSeas, New York, NY USA

**Keywords:** Surgical care, Nepal, Barriers, Affordability, Accessibility, Fear, No trust

## Abstract

**Background:**

Various barriers exist that preclude individuals from undergoing surgical care in low-income countries. Our study assessed the main barriers in Nepal, and identified individuals most at risk for not receiving required surgical care.

**Methods:**

A countrywide survey, using the Surgeons OverSeas Assessment of Surgical Need (SOSAS) survey tool, was carried out in 2014, surveying 2,695 individuals with a response rate of 97%. Our study used data from a subset, namely individuals who required surgical care in the last twelve months. Data were collected on individual characteristics, transport characteristics, and reasons why individuals did not undergo surgical care.

**Results:**

Of the 2,695 individuals surveyed, 207 individuals needed surgical care at least once in the previous 12 months. The main reasons for not undergoing surgery were affordability (*n* = 42), accessibility (*n* = 42) and fear/no trust (*n* = 34). A factor significantly associated with affordability was having a low education (OR = 5.77 of having no education vs. having secondary education). Living in a rural area (OR = 2.59) and a long travel time to a secondary and tertiary health facility (OR = 1.17 and 1.09, respectively) were some of the factors significantly associated with accessibility. Being a woman was significantly associated with fear/no trust (OR = 3.54).

**Conclusions:**

More than half of the individuals who needed surgical care did not undergo surgery due to affordability, accessibility, or fear/no trust. Providing subsidised transport, introducing mobile surgical clinics or organising awareness raising campaigns are measures that could be implemented to overcome these barriers to surgical care.

## Background

Surgical care in low-income countries is limited compared to high-income countries. Countries with middle- and high expenditure account for 30.2% of the world population and had 73.6% of operations in 2004, while poor-expenditure countries accounted for 34.8% of the population but only 3.5% of all surgical procedures [1]. According to a review by Grimes et al., barriers that limit access to surgery in low- and middle-income countries are cultural (acceptability) and structural (accessibility), and most significantly financial (affordability) [2]. Financial barriers include costs for transport [3]. One of the cultural barriers in low-income countries is fear of receiving treatment or undergoing surgery in a health facility, as was shown in studies in Uganda, Kenya and Myanmar [4–6]. Differences between individuals who undergo or who do not undergo surgery exist within a country as well, due to inequalities (e.g. in income) [7]. In addition, most patients in low- and middle-income countries with surgical conditions never reach a health facility [8].

Nepal is a country in South Asia with a population of 26.5 million [9] and a low Human Development Index [10]. Nepal is transitioning from a 10 year internal conflict that ended in 2006, and which led to a government healthcare system unable to meet the needs of the population [11]. Only 43% of the people have access to all-weather roads [12], which limits accessibility to healthcare facilities for a large proportion of the Nepalese population. A study by Weiser et al. imputed the surgical rate in Nepal to be 326 per 100,000 (based on total expenditure on health) [1]. In April 2015, the country was hit by a major earthquake, killing more than 9,000 people and injuring more than 23,000, which worsened Nepal’s already weak healthcare system (with only 2.1 physicians and 50 hospital beds for every 10,000 people) [13], and made access to basic needs, including health care, a priority.

A study by Gupta et al. has described the number of surgical procedures and the unmet surgical need using data collected from a countrywide survey tool, the Surgeons OverSeas Assessment of Surgical Needs (SOSAS) [11]. The aim of our study was to describe the main reasons why individuals did not undergo surgical care in Nepal. In addition, we identified demographic, socio-economic and transport-related characteristics that were associated with these reasons. Our study therefore helps to assess which persons are most at risk for not receiving the surgical care they need, and the reasons why. Also, our study provides suggestions on how to improve access to surgical care of the Nepalese population.

## Methods

The design of this study was a cross-sectional survey of a sample of the Nepalese population. Data were collected between the 25^th^ of May and the 12^th^ of June 2014. Institutional Review Board (IRB) approval was obtained from the Nepal Health Research Council in Kathmandu, Nepal and Nationwide Children’s Hospital in Columbus, Ohio, USA. Verbal informed consent was obtained from all respondents prior to the survey (parental consent, oral assent and/or parental permission were obtained for individuals younger than 18 years). Individuals noted to be cognitively impaired by the household members were excluded from the study; household members of all ages were included. Data were collected confidentially; no personal identifiers were present in the surveys.

### Data collection

The Surgeons OverSeas Assessment of Surgical Needs (SOSAS) is a countrywide survey tool [14]. The different sections of SOSAS are described in Table 1. For the analyses described in this paper, we used the following sections: A) Household information, C) Transportation means, E) General information on the selected respondents, F)-K) health problems (related to surgical care) that respondents experienced, categorised according to six anatomical regions: 1) face, head and neck, 2) chest and breast, 3) abdomen, 4) groin and genitalia, 5) back and 6) extremities. For each anatomical region for which individuals reported health problems, they reported whether they received surgical care or what the main reason was for not receiving surgical care.

**Table 1:** Sections of the Surgeons OverSeas Assessment of Surgical Need (SOSAS)

Section	Section name	Contents	No of questions
Questions for head of household			
A	Household information	Village information	6
B	Living household members	Gender and age of household members	1
C	Transportation means	Time and costs for transport to health facility	15
D	Deceased household members	Demographics and specifics of the deceased	11
Questions for randomly selected household members (two per household)			
E	General information	Demographic and socio-economic information	14
F	Face/head/neck	Health problems and care received of indicated anatomical region	11
G	Chest/breast	Health problems and care received of indicated anatomical region	10
H	Back	Health problems and care received of indicated anatomical region	10
I	Abdomen	Health problems and care received of indicated anatomical region	10
J	Groin/genitalia/buttocks	Health problems and care received of indicated anatomical region	10
K	Extremities	Health problems and care received of indicated anatomical region	12
L	Women’s health	Information on reproduction and menstruation	22

The SOSAS survey in Nepal surveyed a total of 2,695 individuals with a response rate of 97%. Our study sample was drawn from this larger sample. Data were collected using a two-stage randomised cluster sampling in 45 out of 3,915 Village Development Committees (VDCs) in Nepal after stratifying for urban and rural population distribution, and 30 households were sampled per VDC. Details on the larger survey, including the sampling procedure, are described elsewhere [15]. The surveys were conducted by a total of 100 Nepali medical interns and students. Within each household, the head of the household and two randomly selected respondents were interviewed. The surveys were available in English, administered in Nepali and the responses were recorded in English. All data were reviewed daily by the team supervisors to assess for adequacy and accuracy of data collection. Team supervisors would report daily to the overall project supervisor.

### Data description

For our data analysis, we included individuals who needed surgical care in the last twelve months to avoid recall bias that might occur over a longer time period. Three study groups were defined among the individuals who did not undergo surgery. This division was based on the following closed question:

*What was the main reason not to go to a health facility to see a doctor/nurse or not to have an operation or dressings?*

- *No money for health care*
- *No (money for) transportation*
- *No time*
- *Fear/no trust*
- *Not available (facility/personnel/equipment)*
- *No need*

This led to the following study groups:

- Study group 1: *affordability*, consisting of individuals who had no money for health care, or no (money for) transportation;
- Study group 2: *accessibility*, consisting of individuals for whom surgical care was not available (no access to a health facility, personnel or equipment);
- Study group 3: *fear/no trust*, consisting of individuals who did not undergo surgery due to fear/no trust.

We did not include lack of time as a separate study group as it was very small (*n* = 5). Individuals who reported that there was no need for surgery were excluded from the study. The reference group consisted of individuals who underwent surgery. Respondents could belong to more than one study group if they needed surgery more than once.

For the descriptive analyses, we reformulated two variables:

- Occupation was reduced from 7 categories to 3 (unemployed/child, self-employed, salaried);
- Ethnic groups were recategorised from 14 categories to 6, under guidance from a Nepalese national;

For the analyses, we recategorised three additional variables:

- Secondary, tertiary and graduate education were merged as ‘secondary or higher’;
- Transport to a secondary health facility was grouped as motorised and non-motorised transport;
- Transport to a tertiary health facility was grouped as motorised and non-motorised transport.

The categorisation of motorised and non-motorised was chosen, since all modes of motorised transport rely on spending an amount of money (e.g. for fuel, or a ticket) and are suitable for long-distance travel, opposed to non-motorised transport.

As surgical care is provided by secondary or tertiary health facilities, we did not take travel to primary health facilities into account in our analyses.

### Data analysis

Individual characteristics of the study sample were described, and separately for the groups of individuals who did not undergo surgery due to affordability, accessibility, and fear/no trust (study groups 1, 2 and 3, respectively).

Pearson’s chi-square tests and crude odds ratios (cOR) were used to assess the relationship between reason for not undergoing surgery (affordability, accessibility and fear/no trust) and individual characteristics. A logistic regression was carried out to determine the adjusted effect of each individual factor on the reason for not undergoing surgery. A *p*-value of < .05 was considered to be statistically significant, based on two-sided tests. Data were analysed using the software SPSS for Windows (version 20).

## Results

### Barriers for undergoing surgery

Out of 2,695 respondents within SOSAS, 207 individuals (7.7%) were identified who needed surgical care for at least one anatomical location in the last 12 months. No respondent needed surgery more than once on the same anatomical location. The number of respondents within each study group was: 1) affordability, *n* = 42 (20%); 2) accessibility, *n* = 42 (20%); 3) fear/no trust, *n* = 34 (16%). In total, 117 individual respondents (57%) needed surgery, but did not undergo it due to affordability, accessibility, and/or fear/no trust (including one individual who needed surgery twice, but did not undergo it due to two different reasons).

Demographic and socio-economic characteristics of the study sample can be found in Table 2. Educational level was relatively low in the study group ‘affordability’ compared to the complete study sample (57.1% vs. 38.6% without any education). The ethnicity Brahmin/Chhetri was lower (11.9% vs. 30.0%), and Dalit higher (7.1% vs. 2.4%) than in the complete study sample. The study group ‘accessibility’ consisted of a low proportion of individuals living in an urban area compared to the complete study sample (16.7% vs. 29%). The study group ‘fear/no trust’ consisted of a relatively high proportion of women (64.7% vs. 44.9%). Transport-related characteristics of all groups are presented in Table 3. Travel money was less often available for study group 1, and they more often go to a secondary health facility on foot. Travel time, waiting time and costs to secondary and tertiary health facilities were high for study group 2, compared to the complete study sample.

**Table 2:** Description of demographic and socio-economic characteristics of the total study sample, consisting of all individuals that required surgical care in the last 12 months, and the three study groups (no surgical care due to affordability, accessibility and fear/no trust, respectively) separately

Demographic and socio-economic characteristics of the study groups and the total study sample				
	Affordability Study group 1	Accessibility Study group 2	Fear/no trust Study group 3	Complete study sample
	*n* = 42	*n* = 42	*n* = 34	*n* = 207
Male gender %	50.0	50.0	35.3	55.1
Average age (sd)	37.6 (21.0)	36.5 (23.3)	40.7 (21.6)	35.5 (21.0)
Education %				
None	57.1	50.0	41.2	38.6
Primary school	26.2	19.0	17.6	25.6
Secondary school	14.3	21.4	29.4	25.1
Tertiary school	2.4	9.5	5.9	8.7
Graduate degree	0.0	0.0	5.9	1.9
Occupation %				
Unemployed/child^a^	64.3	73.8	72.7	62.2
Self-employed	33.3	23.8	15.2	30.6
Employed	2.4	2.4	12.1	7.3
Ethnic group %				
Janajati	35.7	35.7	35.3	37.2
Brahmin/Chhetri	11.9	31.0	32.4	30.0
Madhesi	11.9	11.9	5.9	8.2
Muslim	4.8	0.0	0.0	3.4
Dalit	7.1	2.4	0.0	2.4
Other	38.6	19.0	26.5	18.8
Living in urban area %	26.2	16.7	32.4	29.0

^a^Occupational status ‘unemployed’ also contains home makers and domestic helpers

**Table 3:** Description of transport-related characteristics of the total study sample, consisting of individuals that required surgical care in the last 12 months, and the three study groups (no surgical care due to affordability, accessibility and fear/no trust, respectively) separately

Transport-related characteristics of the study groups and the total study sample				
	Affordability Study group 1	Accessibility Study group 2	Fear/no trust Study group 3	Complete study sample
	*n* = 42	*n* = 42	*n* = 34	*n* = 207
Secondary health facility				
Transport to health facility^a^ %				
Public transport (bus/taxi)	71.4	83.3	66.7	71.6
Car	0.0	2.4	6.1	2.9
Motorcycle	0.0	0.0	15.2	8.3
Bicycle	2.4	0.0	0.0	1.5
Animal	2.4	0.0	0.0	0.5
On foot	23.8	14.3	12.1	15.2
Travel time to health facility in hours, median (iqr)	1.0 (1.5)	2.5 (10.5)	1.0 (2.7)	1.0 (2.7)
Waiting time for transport in hours, median (iqr)	0.2 (0.4)	1.0 (0.9)	0.1 (0.5)	0.2 (0.5)
Cost for transport in 1000 Nep. Rupees, median (iqr)	0.1 (0.2)	0.4 (1.5)	0.1 (0.6)	0.1 (0.4)
Transport money available %				
Yes	54.8	73.8	57.6	64.2
No	23.8	14.3	9.1	11.3
N/A	21.4	11.9	33.3	24.5
Tertiary health facility				
Transport to health facility^a^ %				
Public transport (bus/taxi)	97.6	95.2	73.5	83.6
Car	0.0	2.4	5.9	2.9
Motorcycle	0.0	0.0	11.8	8.2
On foot	2.4	2.4	8.8	5.3
Travel time to health facility in hours, median (iqr)	2.3 (4.0)	6.0 (17.3)	2.4 (4.7)	2.0 (5.5)
Waiting time for transport in hours, median (iqr)	0.5 (1.0)	1.0 (1.0)	0.3 (1.0)	0.3 (1.0)
Cost for transport in 1000 Nep. Rupees, median (iqr)	0.3 (1.0)	0.8 (3.0)	0.3 (1.0)	0.3 (1.0)
Transport money available %				
Yes	58.5	63.6	61.8	63.8
No	36.6	30.3	17.6	21.4
N/A	4.9	6.1	20.6	14.8

^a^The exact questions were phrased as: what is the main way for you or your household members to go to a secondary/tertiary health facility?

### Individual characteristics associated with not undergoing surgery

Table 4 presents which demographic, socio-economic or transport-related characteristics were significantly associated with belonging to one of the three study groups. The odds were higher for having no education versus secondary education (OR = 5.77 95% CI 2.14-15.58; *p* = .001) for the study group ‘Affordability’. Living in a rural area, having a long travel time to a secondary and tertiary health facility, having high costs for tertiary transport and having no education (versus secondary education) were significantly associated with belonging to the study group ‘accessibility’. Female gender was the only significant characteristic for the study group ‘fear/no trust’ (OR = 3.54 95% CI 1.54-8.15; *p* = .003).

**Table 4:** Characteristics associated with reasons for not undergoing surgery (due to affordability, accessibility and fear/no trust, respectively), using univariate logistic regression. The reference group consisted of individuals who underwent surgery

Characteristics associated with not having surgery							
		Affordability		Accessibility		Fear/no trust	
		Study group 1^a^		Study group 2^a^		Study group 3^a^	
*Household characteristics* ^b^		Crude Odds Ratio^c^ (CI)	*P*-value	Crude Odds Ratio^c^ (CI)	*p*-value	Crude Odds Ratio^c^ (CI)	*P*-value
Rural village type				2.59 (1.03–6.54)	.044		
Travel time to secondary health facility (hours)				1.17 (1.07–1.27)	<.001		
Cost for tertiary transport				1.22 (1.03–1.43)	.021		
Travel time to tertiary health facility (hours)				1.09 (1.03–1.15)	.003		
*Individual characteristics* ^b^							
Female gender						3.54 (1.54–8.15)	.003
Education			.002		.028		
	None	5.77 (2.14–15.58)	.001	2.72 (1.14–6.49)	.024		
	Primary	2.24 (0.77–6.53)	.141	0.88 (0.32–2.41)	.797		
	Secondary or higher	Ref.	Ref.	Ref.	Ref.		

^a^For the dependent variables, the reference group had the value ‘0’, while the study groups had the value ‘1’;

^b^Characteristics that were tested, but did not show any significant results with either of the study groups, were ‘Number of household members’, ‘Motorised transport to secondary health facility’, ‘Cost for secondary transport’, ‘Waiting time for (secondary) transport’, ‘Motorised transport to tertiary health facility’, ‘Waiting time for (tertiary) transport’, and ‘Age’. Non-Motorised transport includes bicycle, animal and on foot, opposed to motorised transport (public and private motorised transport). Ethnicity was not included in the analyses due to the relatively large number of groups. Occupation was not included since it represents socio-economic status, similar to education. Availability of transport money was not included due to the relatively large size of the group ‘not applicable’;

^c^Only statistically significant odds ratios are presented in the table

## Discussion

Our results show that more than half of the people (57%) in our study that needed surgery did not receive it due to affordability, accessibility, or fear/no trust in the last twelve months. This figure indicates a low number of surgical procedures by population in Nepal. This is confirmed by a study that imputed the surgical rate per 100,000 in Nepal to be 326, which is lower than in Western-European countries such as France (13,667), the UK (13,635) and Germany (9,331) [1], where financial barriers and barriers related to accessibility are much lower, but comparable to other Asian countries such as Pakistan (411), Bangladesh (192) and Tajikistan (181) [1].

Several transport-related characteristics were more prevalent in the study group that did not undergo surgery due to affordability, e.g. not having transport money available and going to a secondary health facility by foot (Tables 2 and 3). This implies that individuals with lower financial means have more problems in reaching a health facility than other individuals. The hypothesis that this group has less money to spend is supported by the fact that it consists of a large proportion of individuals without education, which can be considered an indicator for socio-economic status. One way to overcome this inequality is to provide free or subsidised transport for patients who need surgical care but cannot afford transport costs, which has before been suggested in another setting in Ethiopia [16].

Transport-related characteristics were also significantly associated with having an accessibility barrier for undergoing surgical care (e.g. a longer travel time and higher costs for transport). This implies that, even if there are sufficient numbers of surgical personnel, around one in five individuals (20%) will not reach them. Living in a rural area was also significantly associated with this group (Table 4), which has been identified as a barrier in other studies as well [3, 17, 18]. Mobile family planning clinics have been effective in performing vasectomies in hard-to-reach areas in Nepal [19]. Although this is only one procedure, it suggests a similar approach could be used for other surgical procedures to improve accessibility.

Women more often had fear/no trust as a barrier for not undergoing surgery than men. Caesarean section is a crucial operation for women, e.g. in Tribhuvan University Teaching Hospital in Kathmandu, Nepal, the rate was 25.4% [20]. A review by Grimes et al. describes that in some cultures childbirth is seen as a natural event [2], and a difficult birth carries the stigma of having a defective body [21] or being the result of infidelity or an extramarital affair [22, 23]. Although not specified within our questionnaire, similar reasons could be the foundation of the results found within this study. In addition, although there has been a recent increase in the number of female medical students, healthcare in Nepal is currently mostly provided by men [24], which might also contribute to fear/no trust in women. Raising awareness among women on the importance of surgical care is a potential solution to overcome this issue. Another solution could be to increase the proportion of female practitioners and female health workers.

Finally, the recent major earthquake that hit Nepal aggravated access to surgical care in two ways. First, there has been a sharp increase in surgical need, with more than 23,000 people injured on top of the normal case load [13]. Second, hospitals and health centres have been damaged by the earthquake, which decreases access especially in remote locations. A health infrastructure damage assessment is currently ongoing [25].

### Limitations

Several limitations exist in our study. First, we had limited socio-economic data on our respondents, e.g. we lacked household income. In the analyses we used educational level as an indicator for socio-economic status, but income would be a more exact measure to assess whether there is really a difference in financial means between study group 1 and the reference group.

The SOSAS tool has thus far mainly been used to describe the surgical disease burden. The identification of barriers to care as a main objective by using this tool is relatively novel, and should be validated in other contexts. An improvement of the SOSAS tool was made when it was implemented in Nepal, namely the addition of a visual physical examination by a medical professional [11]. We feel that this combined methodology, and the high agreement between the physical examination and the verbal response (94.6%), increases reliability of the self-reported answers in the survey. However, chest and groin examinations were excluded from this examination. Due to the sensitive nature of the anatomical region groin/genitalia/buttocks, there might have been an underrepresentation in the number of reported problems for this region. For identifying the main reasons why individuals did not undergo surgery, a closed question was used, which might have limited the number of potential reasons. A qualitative approach would have given more detailed insight in these reasons.

Only 15 of 75 districts were sampled. Districts were selected proportional to population, so districts that had larger and more populous cities had a greater chance of being selected. Since accessibility is more often seen as a barrier to surgical care in individuals from rural areas (Table 2), the results from this study are likely to be an underestimation when it comes to the actual proportion of individuals who did not receive surgical care due to accessibility issues.

In general, respondents are prone to recall bias for events that happened a long time ago. To reduce this bias, we only included health events within the last 12 months. A study on recall of injuries showed that a period of up to twelve months can safely be used for important events [26].

Since the required sample size was not calculated to answer this research question, this might have been the reason that not more significant odds ratios have been identified. For that reason, we did not rely only on significance but also included the proportions of all characteristics for the different study groups in Tables 2 and 3.

## Conclusions

Our results from the Nepal SOSAS study show that more than half of the respondents do not undergo surgery due to affordability, accessibility, or fear/no trust. Individuals from rural areas, with longer travel times for health facilities, higher travel costs, lower education, and women, face on average more barriers for undergoing surgical care than individuals who do not belong to these groups. Providing subsidised transport for individuals who cannot afford transport costs might aid in overcoming this barrier. Another solution is the introduction of mobile surgical clinics. Since women more often do not undergo surgery due to fear/no trust, the surgical rate can be improved by specifically addressing this group with awareness raising campaigns or increasing the proportion of female practitioners and female health workers.

## Abbreviations

IRB: Institutional Review Board
SOSAS: Surgeons OverSeas Assessment of Surgical Needs
VDC: Village Development Committee
